# Disparities in Telemedicine Utilization among Kidney Transplant Candidates and Recipients

**DOI:** 10.34067/KID.0000001308

**Published:** 2026-07-10

**Authors:** Jae-Hyung Chang, Alexander Moon, Kundan R. Jana, Adarsh Ramakrishnan, Jennifer J. van Bever, Demetra S. Tsapepas, Sumit Mohan, Syed Ali Husain

**Affiliations:** 1 Division of Nephrology, Department of Medicine, Columbia University Vagelos College of Physicians and Surgeons, New York, New York; 2 Emory College, Atlanta, Georgia; 3 New York-Presbyterian Hospital, New York, New York; 4 Department of Epidemiology, Columbia University Mailman School of Public Health, New York, New York; 5 Columbia University Renal Epidemiology (CURE) Group, New York, New York

**Keywords:** health equity, diversity, and inclusion, health policy, kidney transplantation

## Introduction

Telemedicine has the potential to improve health care access for patients with ESKD.^1–3^ Medicare-insured patients without geographic restrictions are at a risk of losing access to telehealth services because of failure to durably extend telehealth coverage flexibility.^4^

## Methods

Outpatient records from our transplantation center were reviewed. All adults (≥18 years) scheduled for at least one telemedicine visit with a physician for pretransplant evaluation or posttransplant care between January 1, 2022, and June 30, 2023, were included. Visit completion was defined as patient logging in and completing the entire visit.

The primary outcome was completion of all scheduled telemedicine visits. Characteristics of patients who completed all visits were compared with those who missed one or more visits using chi-squared tests for categorical variables and Wilcoxon rank-sum tests for continuous variables. Multivariable logistic regression was used to assess factors associated with completion of all scheduled telemedicine visits. Subgroup analyses were performed separately for pretransplant and posttransplant patients. All statistical analyses were performed using *R* version 4.2.2. The study was approved by the Columbia University Institutional Review Board.

## Results

A total of 3277 patients (mean age 55±15 years, 58% male) were scheduled for at least one virtual visit during the study period. Of them, 1124 patients (34%) completed all scheduled visits (Supplemental Tables 1 and 2). Patients who completed all visits were more likely to be White (45% versus 37%; *P* < 0.001) and employed (22% versus 20%; *P* = 0.04) compared with those who did not complete all visits (Supplemental Table 3). In multivariable analysis, older age (≥60 years; adjusted odds ratio [adj. OR], 1.53; 95% confidence interval [CI], 1.10 to 2.13; *P* = 0.01) and Medicaid (versus Medicare) insurance (adj. OR, 1.78; 95% CI, 1.04 to 3.00; *P* = 0.03) were independently associated with completion of all virtual visits (pretransplant and/or post-transplant). Sex, ethnicity, preferred language, employment, and education level were not significantly associated with visit completion. Among pretransplant candidates, those aged ≥60 years were more likely to complete all scheduled visits (adj. OR, 2.04; 95% CI, 1.18 to 3.60; *P* = 0.01), whereas Black candidates were less likely to do so compared with White candidates (adj. OR, 0.58; 95% CI, 0.34 to 0.96; *P* = 0.03). No factors were significantly associated with posttransplant visit completion among transplant recipients (Figure 1).

**Figure 1:**
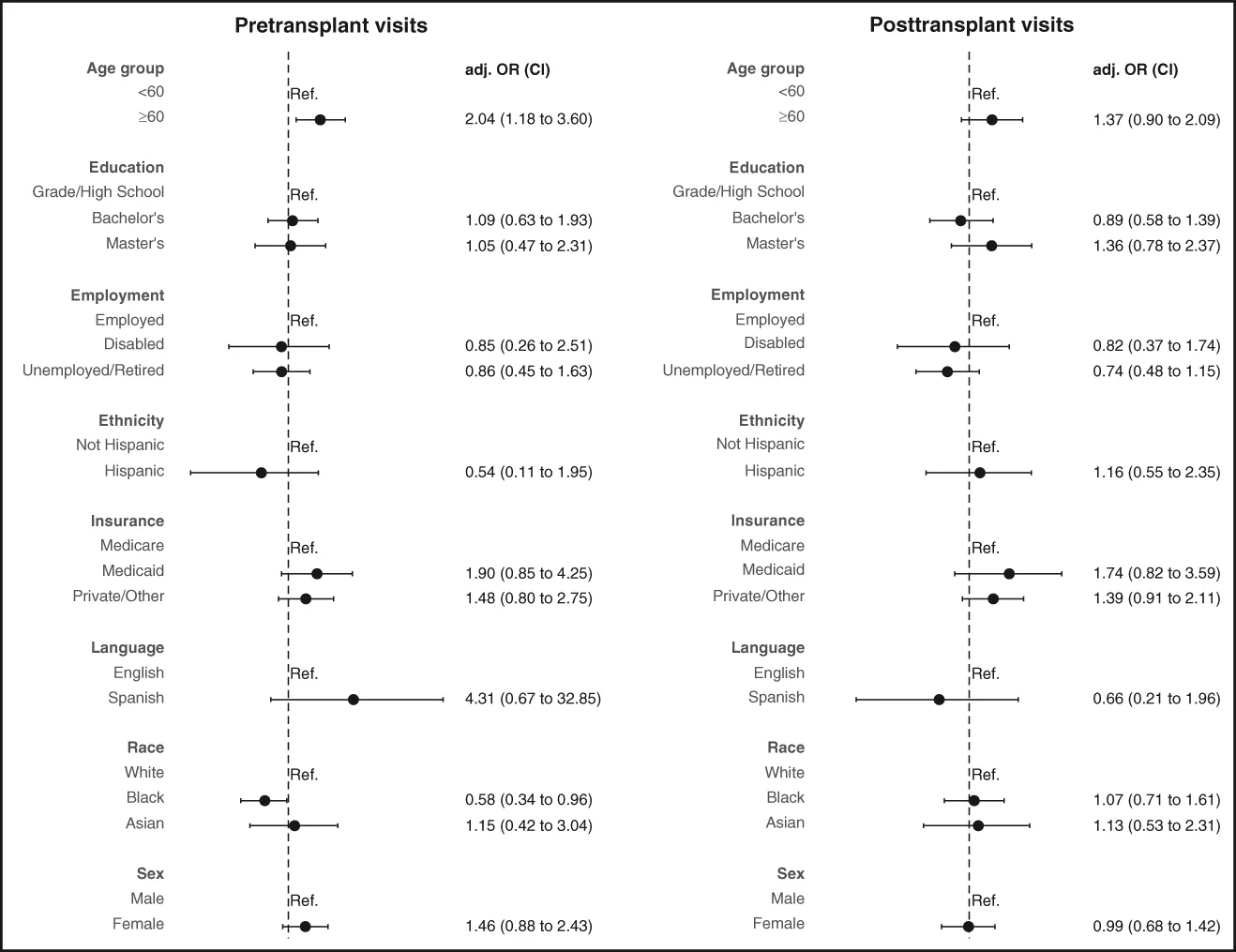
**Forest plot of multivariate regression analysis showing characteristics associated with completion of pretransplant and post-transplant visits.** adj. OR, adjusted odds ratio; CI, confidence intervals; Ref., reference group.

## Discussion

Kidney transplant candidates and recipients face geographic, logistical, and financial barriers to care.^1^ A recent national registry study showed that 85% of kidney transplant recipients reside in nonrural areas, with higher concentration in states such as New York and California.^5^ They concluded that restricting telemedicine to rural areas risks exacerbating existing racial and ethnic disparities in kidney transplantation as Black, Hispanic, and Asian recipients are disproportionately concentrated in these nonrural areas.

We found age and racial disparities in successful telemedicine utilization among kidney transplant candidates undergoing a pretransplant evaluation at our center. This result is consistent with previous reports that Black race is associated with lower telemedicine use.^2^ Although previous studies have demonstrated that older age is independently associated with lower telemedicine use in general, possibly because of lower use of digital health technology, our study showed that older candidates aged ≥60 years were more likely to complete all virtual pretransplant evaluation appointments.^2^ In a qualitative study involving 60 interviews conducted among patients aged ≥70 years with advanced CKD, older patients described telehealth as more convenient, less costly, and more efficient than clinic visits.^6^ In this study, lower satisfaction was associated with telehealth among older patients with lower socioeconomic status and among patients identifying as Black, Hispanic, and Native American. Although our observation may be, at least in part, due to selection bias, limited mobility, fear for infection, and greater availability among those retired may have contributed to our findings of high completion rate of virtual visits among older candidates. Once transplanted, there was no difference in completion rates of virtual appointments, suggesting that successful posttransplant follow-up care can be achieved using telemedicine across all age groups and races—perhaps because of the selection bias of patient who make it through the evaluation and waiting process to transplantation. Although completion rates may be structurally confounded by the number of scheduled visits, our study reflects real-world practice, where many patients have only one video visit within a care model that combines in-person and virtual appointments. As the study cohort was evaluated during the coronavirus disease 2019 global public health emergency, our findings may have limited generalizability.

In a retrospective study conducted at the Nashville Veterans Affairs hospital, the timeliness to kidney transplant waitlist evaluation improved markedly with addition of telehealth, hence, increasing access to transplant.^7^ Our findings suggest that the widespread adoption of telemedicine benefits patients with ESKD, specifically older kidney transplant candidates, and supports passing of more comprehensive and permanent laws to provide long-term certainty and avoid disruption in access to virtual care for everyone without location restrictions. Our study also underscores the risk of unintended consequences of exacerbating racial disparities in access to kidney transplantation in the absence of active monitoring.

## Supplementary Material

## Data Availability

Original data generated for the study will be made available upon reasonable request to the corresponding author. Data Type: Health Care Data; Aggregated Data. Reason for Restricted Access: Patient data.

## Declarative Statements

This study includes clinical experimentation and received Institutional Review Board or Ethics Committee approval. All patients provided written informed consent. This study includes clinical experimentation and complies with the Declaration of Helsinki. The clinical and research activities being reported are consistent with the Principles of the Declaration of Istanbul as outlined in the Declaration of Istanbul on Organ Trafficking and Transplant Tourism.

## Supplemental Material

Supplemental Table 1. Number of telemedicine visits per patient.

Supplemental Table 2. Number of patients by completion rate of scheduled telemedicine visits.

Supplemental Table 3. Comparison of baseline patient demographics based on completion of all scheduled telemedicine visits.
