# Supplementary material for: Disparities in Telemedicine Utilization among Kidney Transplant Candidates and Recipients
Source: Kidney360. 2026 Jul 10;7(9):2128–30. doi: 10.34067/KID.0000001308 (PMC13641446; doi:10.34067/KID.0000001308)
Supplement: Supplementary file 1 [file kidney360-7-2128-s001.pdf]

ASN American Society of Nephrology
JASN Journal of the American Society of Nephrology
KIDNEY360 Accessing Our World From Every Angle
CJASN Clinical Journal of the American Society of Nephrology

ASN Journal Disclosure Form

As per ASN journal policy, I have disclosed any financial relationships or commitments I have held in the past 36 months as included below. I have listed my Current Employer below to indicate there is a relationship requiring disclosure. If no relationship exists, my Current Employer is not listed.

J. Chang reports the following:
Employer: Columbia University

I understand that the information above will be published within the journal article, if accepted, and that failure to comply and/or to accurately and completely report the potential financial conflicts of interest could lead to the following: 1) Prior to publication, article rejection, or 2) Post-publication, sanctions ranging from, but not limited to, issuing a correction, reporting the inaccurate information to the authors' institution, banning authors from submitting work to ASN journals for varying lengths of time, and/or retraction of the published work.

Name: Jae Hyung Chang
Manuscript ID: K360-2026-000050R1
Manuscript Title: Disparities in Telemedicine Utilization Among Kidney Transplant Candidates and Recipients
Date of Completion: May 6, 2026
Disclosure Updated Date: May 6, 2026

ASN American Society of Nephrology | JASN Journal of the American Society of Nephrology | KIDNEY360 Accessing Our World From Every Angle | CJASN Clinical Journal of the American Society of Nephrology

ASN Journal Disclosure Form

As per ASN journal policy, I have disclosed any financial relationships or commitments I have held in the past 36 months as included below. I have listed my Current Employer below to indicate there is a relationship requiring disclosure. If no relationship exists, my Current Employer is not listed.

S. Husain reports the following:
Consultancy: Novartis (Relationship ended on 10/2/2025)

I understand that the information above will be published within the journal article, if accepted, and that failure to comply and/or to accurately and completely report the potential financial conflicts of interest could lead to the following: 1) Prior to publication, article rejection, or 2) Post-publication, sanctions ranging from, but not limited to, issuing a correction, reporting the inaccurate information to the authors' institution, banning authors from submitting work to ASN journals for varying lengths of time, and/or retraction of the published work.

Name: Syed Ali Husain
Manuscript ID: K360-2026-000050R1
Manuscript Title: Disparities in Telemedicine Utilization Among Kidney Transplant Candidates and Recipients
Date of Completion: May 11, 2026
Disclosure Updated Date: March 5, 2026

ASN American Society of Nephrology | JASN Journal of the American Society of Nephrology | KIDNEY360 Accessing Our World From Every Angle | CJASN Clinical Journal of the American Society of Nephrology

ASN Journal Disclosure Form

As per ASN journal policy, I have disclosed any financial relationships or commitments I have held in the past 36 months as included below. I have listed my Current Employer below to indicate there is a relationship requiring disclosure. If no relationship exists, my Current Employer is not listed.

K. Jana reports the following:
Employer: Columbia University

I understand that the information above will be published within the journal article, if accepted, and that failure to comply and/or to accurately and completely report the potential financial conflicts of interest could lead to the following: 1) Prior to publication, article rejection, or 2) Post-publication, sanctions ranging from, but not limited to, issuing a correction, reporting the inaccurate information to the authors' institution, banning authors from submitting work to ASN journals for varying lengths of time, and/or retraction of the published work.

Name: Kundan Jana
Manuscript ID: K360-2026-000050R1
Manuscript Title: Disparities in Telemedicine Utilization Among Kidney Transplant Candidates and Recipients
Date of Completion: May 11, 2026
Disclosure Updated Date: May 11, 2026

ASN Journal Disclosure Form

As per ASN journal policy, I have disclosed any financial relationships or commitments I have held in the past 36 months as included below. I have listed my Current Employer below to indicate there is a relationship requiring disclosure. If no relationship exists, my Current Employer is not listed.

S. Mohan reports the following:
Consultancy: Sanofi (Relationship has not ended); DEKA Research (Relationship has not ended); HSAG (Relationship ended on 8/1/2025); International Society of Nephrology (Relationship has not ended); Viscera (Relationship has not ended); Specialist Direct (Relationship has not ended); Takeda (Relationship has not ended); Viscera (Relationship has not ended)

I understand that the information above will be published within the journal article, if accepted, and that failure to comply and/or to accurately and completely report the potential financial conflicts of interest could lead to the following: 1) Prior to publication, article rejection, or 2) Post-publication, sanctions ranging from, but not limited to, issuing a correction, reporting the inaccurate information to the authors' institution, banning authors from submitting work to ASN journals for varying lengths of time, and/or retraction of the published work.

Name: Sumit Mohan
Manuscript ID: K360-2026-000050R1
Manuscript Title: Disparities in Telemedicine Utilization Among Kidney Transplant Candidates and Recipients
Date of Completion: May 11, 2026
Disclosure Updated Date: May 11, 2026

ASN American Society of Nephrology
JASN Journal of the American Society of Nephrology
KIDNEY360 Accessing Our World From Every Angle
CJASN Clinical Journal of the American Society of Nephrology

ASN Journal Disclosure Form

As per ASN journal policy, I have disclosed any financial relationships or commitments I have held in the past 36 months as included below. I have listed my Current Employer below to indicate there is a relationship requiring disclosure. If no relationship exists, my Current Employer is not listed.

A. Moon has nothing to disclose.

I understand that the information above will be published within the journal article, if accepted, and that failure to comply and/or to accurately and completely report the potential financial conflicts of interest could lead to the following: 1) Prior to publication, article rejection, or 2) Post-publication, sanctions ranging from, but not limited to, issuing a correction, reporting the inaccurate information to the authors' institution, banning authors from submitting work to ASN journals for varying lengths of time, and/or retraction of the published work.

Name: Alexander Moon
Manuscript ID: K360-2026-000050R1
Manuscript Title: Disparities in Telemedicine Utilization Among Kidney Transplant Candidates and Recipients
Date of Completion: May 14, 2026
Disclosure Updated Date: May 14, 2026

ASN American Society of Nephrology
JASN Journal of the American Society of Nephrology
KIDNEY360 Accessing Our World From Every Angle
CJASN Clinical Journal of the American Society of Nephrology

ASN Journal Disclosure Form

As per ASN journal policy, I have disclosed any financial relationships or commitments I have held in the past 36 months as included below. I have listed my Current Employer below to indicate there is a relationship requiring disclosure. If no relationship exists, my Current Employer is not listed.

A. Ramakrishnan reports the following:
Employer: NewYork-Presbyterian

I understand that the information above will be published within the journal article, if accepted, and that failure to comply and/or to accurately and completely report the potential financial conflicts of interest could lead to the following: 1) Prior to publication, article rejection, or 2) Post-publication, sanctions ranging from, but not limited to, issuing a correction, reporting the inaccurate information to the authors' institution, banning authors from submitting work to ASN journals for varying lengths of time, and/or retraction of the published work.

Name: Adarsh Ramakrishnan
Manuscript ID: K360-2026-000050R1
Manuscript Title: Disparities in Telemedicine Utilization Among Kidney Transplant Candidates and Recipients.
Date of Completion: May 6, 2026
Disclosure Updated Date: May 6, 2026

ASN American Society of Nephrology | JASN Journal of the American Society of Nephrology | KIDNEY360 Accessing Our World From Every Angle | CJASN Clinical Journal of the American Society of Nephrology

ASN Journal Disclosure Form

As per ASN journal policy, I have disclosed any financial relationships or commitments I have held in the past 36 months as included below. I have listed my Current Employer below to indicate there is a relationship requiring disclosure. If no relationship exists, my Current Employer is not listed.

D. Tsapepas has nothing to disclose.

I understand that the information above will be published within the journal article, if accepted, and that failure to comply and/or to accurately and completely report the potential financial conflicts of interest could lead to the following: 1) Prior to publication, article rejection, or 2) Post-publication, sanctions ranging from, but not limited to, issuing a correction, reporting the inaccurate information to the authors' institution, banning authors from submitting work to ASN journals for varying lengths of time, and/or retraction of the published work.

Name: Demetra Tsapepas
Manuscript ID: K360-2026-000050R1
Manuscript Title: Disparities in Telemedicine Utilization Among Kidney Transplant Candidates and Recipients
Date of Completion: June 23, 2026
Disclosure Updated Date: June 23, 2026

ASN Journal Disclosure Form

As per ASN journal policy, I have disclosed any financial relationships or commitments I have held in the past 36 months as included below. I have listed my Current Employer below to indicate there is a relationship requiring disclosure. If no relationship exists, my Current Employer is not listed.

J. Van Bever has nothing to disclose.

I understand that the information above will be published within the journal article, if accepted, and that failure to comply and/or to accurately and completely report the potential financial conflicts of interest could lead to the following: 1) Prior to publication, article rejection, or 2) Post-publication, sanctions ranging from, but not limited to, issuing a correction, reporting the inaccurate information to the authors' institution, banning authors from submitting work to ASN journals for varying lengths of time, and/or retraction of the published work.

Name: Jennifer Van Bever
Manuscript ID: K360-2026-000050R1
Manuscript Title: Disparities in Telemedicine Utilization Among Kidney Transplant Candidates and Recipients
Date of Completion: June 25, 2026
Disclosure Updated Date: June 23, 2026
