# Supplementary material for: Disparities in Telemedicine Utilization among Kidney Transplant Candidates and Recipients
Source: Kidney360. 2026 Jul 10;7(9):2128–30. doi: 10.34067/KID.0000001308 (PMC13641446; doi:10.34067/KID.0000001308)
Supplement: Supplementary file 2 [file kidney360-7-2128-s002.pdf]

Supplemental Table 1. Number of telemedicine visits per patient

	**Median**	**IQR**
**Number of total visits per patient**	3	2, 5
**Number of 100% completed total visits per patient**	2	1, 3
**Number of total pre-transplant visits per patient**	4	3, 6
**Number of 100% completed pre-transplant visits per patient**	3	2, 3
**Number of total post-transplant visits per patient**	2	1, 4
**Number of 100% completed post-transplant visits per patient**	1	1, 2

Supplemental Table 2. Number of patients by completion rate of scheduled telemedicine visits

Percentage of visits completed	Pre-transplant	Post-transplant	Total
100%	463	661	1124
75-99%	171	130	301
50-74%	378	613	991
25-49%	191	259	450
1-24%	62	34	96
0%	120	195	315

Supplemental Table 3. Comparison of baseline patient demographics based on completion of all scheduled telemedicine visits

	Did not complete all visits	Completed all visits	All	p-value
N	2153 (66%)	1124 (34%)	3277 (100%)	
**Age**				0.24
Mean (SD)	54 (15)	55 (15)	55 (15)	
Median (IQR)	57 (44, 66)	57 (45, 66)	57 (44, 66)	
**Age category**				0.20
<60	1291 (60%)	648 (58%)	1939 (59%)	
≥60	862 (40%)	476 (42%)	1338 (41%)	
**Sex**				0.29
Male	1267 (59%)	640 (57%)	1907 (58%)	
Female	886 (41%)	484 (43%)	1370 (42%)	
**Race**				<0.001
Asian	103 (5%)	48 (4%)	151 (5%)	
African American	541 (25%)	234 (21%)	775 (23%)	
White	794 (37%)	505 (45%)	1299 (40%)	
Other/unknown	715 (33%)	337 (30%)	1052 (32%)	
**Ethnicity**				0.56
Hispanic or Latino	561 (26%)	286 (25%)	847 (26%)	
Not Hispanic or Latino	1398 (65%)	749 (67%)	2147 (65%)	
Declined/unknown	194 (9%)	89 (8%)	283 (9%)	
**Preferred language**				0.96
English	1627 (76%)	851 (76%)	2478 (76%)	
Spanish	302 (14%)	157 (14%)	459 (14%)	
Other/unknown	224 (10%)	116 (10%)	340 (10%)	
**Insurer**				0.16
Medicaid	345 (16%)	149 (13%)	494 (15%)	
Medicare	966 (45%)	474 (42%)	1440 (44%)	
Private/other	746 (35%)	401 (36%)	1147 (35%)	
Unknown	96 (4%)	100 (9%)	196 (6%)	
**Employment**				0.04
Employed	422 (20%)	246 (22%)	668 (20%)	
Unemployed/retired	876 (41%)	397 (35%)	1273 (39%)	
Disabled	135 (6%)	61 (6%)	196 (6%)	
Unknown	720 (33%)	420 (37%)	1140 (35%)	
**Transplant status**				0.15
Not transplanted	732 (34%)	357 (32%)	1089 (33%)	

Transplanted	1184 (55%)	649 (58%)	1833 (56%)	
Unknown	237 (11%)	118 (10%)	355 (11%)	
**New vs. follow up**				0.20
New patient	801 (37%)	444 (40%)	1245 (38%)	
Follow up	1352 (63%)	680 (60%)	2032 (62%)	
**Pre- vs. post-transplant visit**				0.37
Pre	922 (43%)	463 (41%)	1385 (42%)	
Post	1231 (57%)	661 (59%)	1892 (58%)	
**Highest education**				0.39
High school	567 (26%)	300 (27%)	867 (26%)	
Bachelor's degree	729 (34%)	409 (36%)	1138 (35%)	
Postgraduate	203 (9%)	129 (12%)	332 (10%)	
Unknown	654 (31%)	286 (25%)	940 (29%)	
